# Inducible Bronchus–Associated Lymphoid Tissue (iBALT) Attenuates Pulmonary Pathology in a Mouse Model of Allergic Airway Disease

**DOI:** 10.3389/fimmu.2020.570661

**Published:** 2020-09-25

**Authors:** Ji Young Hwang, Aaron Silva-Sanchez, Damian M. Carragher, Maria de la Luz Garcia-Hernandez, Javier Rangel–Moreno, Troy D. Randall

**Affiliations:** ^1^Division of Clinical Immunology and Rheumatology, The Department of Medicine, University of Alabama at Birmingham, Birmingham, AL, United States; ^2^Department of Microbiology and Immunology, University of Rochester, Rochester, NY, United States; ^3^The Trudeau Institute, Saranac Lake, NY, United States; ^4^Division of Allergy Immunology and Rheumatology, The Department of Medicine, University of Rochester, Rochester, NY, United States

**Keywords:** inducible bronchus associated lymphoid tissue, asthma, pulmonary inflammation, ectopic lymphoid tissue, pulmonary allergy

## Abstract

Inducible Bronchus Associated Lymphoid Tissue (iBALT) is an ectopic lymphoid tissue associated with severe forms of chronic lung diseases, including chronic obstructive pulmonary disease, rheumatoid lung disease, hypersensitivity pneumonitis and asthma, suggesting that iBALT may exacerbate these clinical conditions. However, despite the link between pulmonary pathology and iBALT formation, the role of iBALT in pathogenesis remains unknown. Here we tested whether the presence of iBALT in the lung prior to sensitization and challenge with a pulmonary allergen altered the biological outcome of disease. We found that the presence of iBALT did not exacerbate Th2 responses to pulmonary sensitization with ovalbumin. Instead, we found that mice with iBALT exhibited delayed Th2 accumulation in the lung, reduced eosinophil recruitment, reduced goblet cell hyperplasia and reduced mucus production. The presence of iBALT did not alter Th2 priming, but instead delayed the accumulation of Th2 cells in the lung following challenge and altered the spatial distribution of T cells in the lung. These results suggest that the formation of iBALT and sequestration of effector T cells in the context of chronic pulmonary inflammation may be a mechanism by which the immune system attenuates pulmonary inflammation and prevents excessive pathology.

## Introduction

Inducible Bronchus Associated Lymphoid Tissue (iBALT) is an ectopic lymphoid tissue that forms in the lung following inflammation or infection (1, 2). Large B cell follicles that often contain germinal centers (GCs) and a dense network of follicular dendritic cells (FDCs) are the most prominent features of iBALT (3). Unlike conventional lymph nodes, iBALT is not encapsulated, but is instead embedded in the lung tissue, most often along large bronchi or filling the peri-vascular space of pulmonary artieries (4, 5). Despite being in a mucosal tissue, iBALT often lacks a well-defined M cell-containing dome epithelium (6) and its high endothelial venules (HEVs) express peripheral lymph node addressin (PNAd) (7) rather than mucosal addressin cell adhesion molecule (MAdCAM). Thus, although iBALT clearly participates in pulmonary mucosal immune responses, it lacks some of the features often associated with classic mucosal lymphoid organs.

Unlike conventional lymphoid organs, which develop independently of antigen during embryogenesis (8, 9), iBALT forms following pulmonary infection or inflammation (10, 11). Although iBALT may develop at any time after birth given the proper pulmonary insult, it most easily forms in neonatal mice due to the relative paucity of Tregs and the relative abundance of IL-17-producing γδT cells (12–14). Similarly, infant humans also seem to develop (or maintain) iBALT more easily than adults, as the frequency of iBALT areas in the lungs of healthy subjects progressively declines with age (15, 16). Interestingly, the neonatal period is the same developmental window when microbial exposure, or lack thereof, imprints the immune system to be more or less susceptible to developing atopic responses to foreign antigens (17, 18) or developing autoreactive responses to self antgens—the so—called hygiene effect.

Given that it functions as a lymphoid tissue (19), it is not too surprising that iBALT plays an important role in immunity to pulmonary infections. For example, mice lacking conventional lymphoid organs develop iBALT following infection with influenza and are able to resist doses of virus that would normally be lethal to mice lacking iBALT (19). Conversely, mice lacking the chemokines that promote iBALT formation succumb to much lower doses of influenza virus (7). Those same chemokines are essential for the proper formation of pulmonary granulomas, the recruitment of antigen-specific T cells to the lung and the development of protective immunity to *Mycobacterium tuberculosis* (20, 21). Moreover, the pulmonary administration of inflammatory agents that trigger iBALT formation also leads to enhanced resistance to influenza, SARS corona virus, pneumovirus, *Francicella tulerensis* and *Coxiella burnetti* (12, 19, 22–24). Thus, the presence of iBALT is clearly beneficial in the context of pulmonary infections.

In contrast, iBALT is a feature of pulmonary pathology that is associated with a variety of chronic lung diseases, including chronic obstructive pulmonary disease (COPD) (25, 26), rheumatoid lung disease (3), hypersensitivity pneumonitis (27), and asthma (28, 29), suggesting that iBALT may exacerbate these clinical conditions. For example, COPD patients make autoantibodies that react with pulmonary antigens (30) and patients with advanced disease have reactive iBALT areas with well-developed GCs that show evidence of antigen-driven selection (31). Similarly, patients with rheumatoid lung disease develop large GC-containing areas of iBALT that are surrounded by plasma cells that produce autoantibodies (3). Thus, many investigators have concluded that the presence of iBALT contributes to pathology in the context of chronic inflammatory diseases. However, it is not clear whether iBALT is a cause or consequence of pulmonary inflammation or whether it contributes in any way to pulmonary pathology (32).

Given the link between iBALT and pulmonary pathology, we tested whether pre-existing iBALT would affect acute allergic responses to a pulmonary allergen. Surprisingly, we found that the presence of iBALT did not exacerbate allergic responses to repeated pulmonary sensitization with ovalbumin (OVA). Instead, mice with iBALT had reduced Th2-associated mRNA expression, less eosinophil recruitment to the lungs and airways, attenuated goblet cell hyperplasia and reduced mucus production following pulmonary sensitization and challenge with OVA. Interestingly, the presence of iBALT did not alter the initial priming of Th2 cells, but instead delayed their recruitment to the lung and altered their spatial distribution following challenge—Th2 cells were preferentially sequestered in iBALT, which reduced the numbers of Th2 cells in the lung parenchyma. These results suggest that the formation of iBALT and sequestration of effector T cells in the context of pulmonary inflammation may be a mechanism by which the immune system attenuates pulmonary inflammation and prevents excessive pathology.

## Results

### The Presence of iBALT Prior to Sensitization Ameliorates Allergen–Induced Pathology

**Figure 1:**
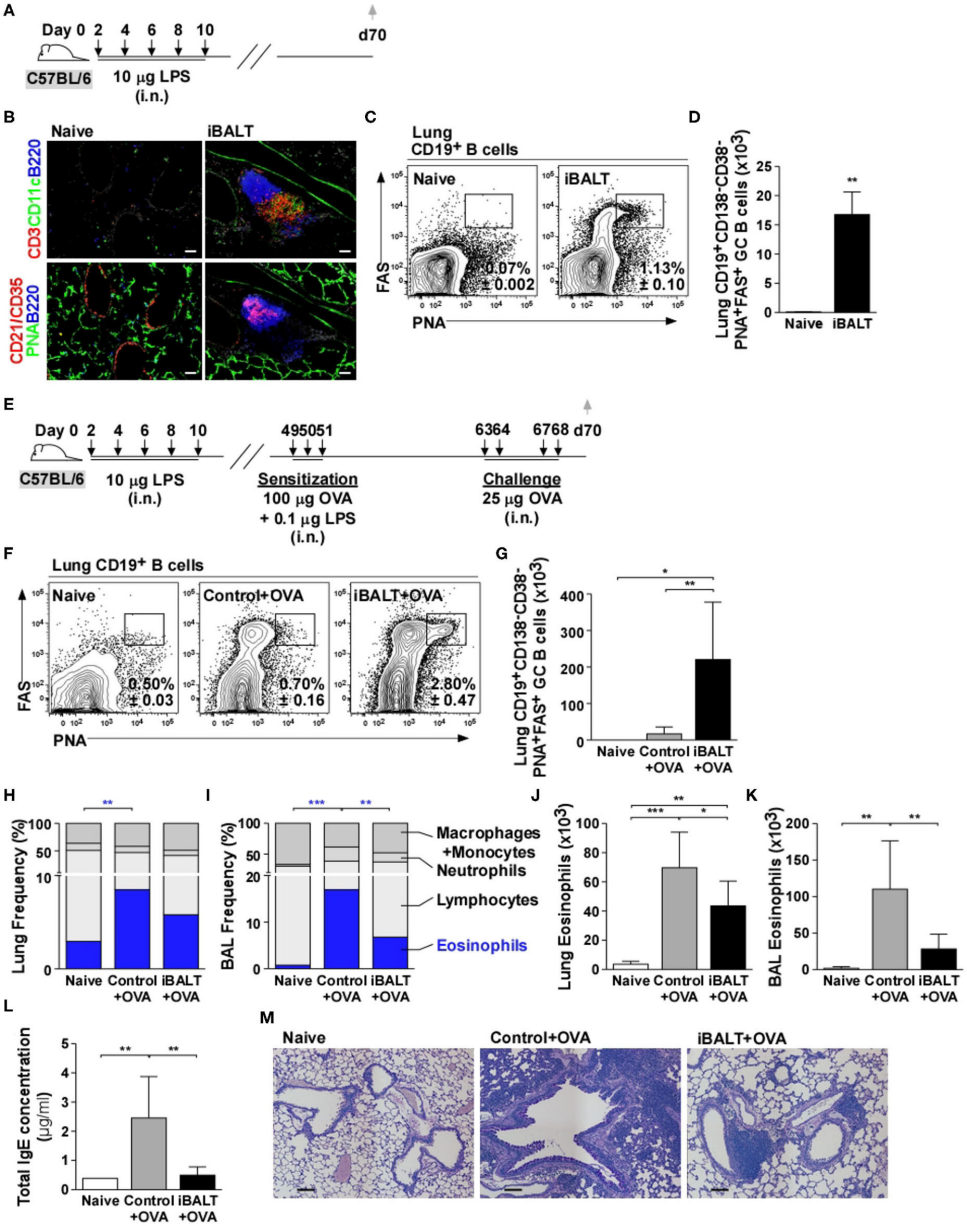
The presence of iBALT reduces OVA–induced eosinophilia and pulmonary pathology. **(A)** Timing of iBALT induction and analysis. **(B)** We probed cryosections of lungs with antibodies against CD3, CD11c, B220, CD21/35 and PNA (scale bar = 100 μm). **(C,D)** The frequencies **(C)** and numbers **(D)** of CD19^+^CD138^−^CD38^−^PNA^+^FAS^+^ GC B cells in the lung were determined by flow cytometry. **(E)** Timing of iBALT induction, allergic sensitization and antigen challenge and analysis. **(F,G)** The frequencies **(F)** and numbers **(G)** of CD19^+^CD138^−^CD38^−^PNA^+^FAS^+^ GC B cells were determined by flow cytometry. **(H–K)** Differential cell counts were determined by cytospin and the proportion of eosinophils in the lung and BAL are shown as frequencies **(H,I)** and numbers **(J,K)**. **(L)** Total serum IgE concentration was measured by ELISA. **(M)** PAS staining of paraffin–embedded lung sections (scale bar = 100 μm). All data show mean ± SD of 4–5 mice per group; *P < 0.05, **P < 0.01, ***P < 0.001. Experiments were performed 5 times **(A–D)** or 4 times **(E–M)**.

To test the role of iBALT in a mouse model of allergic lung inflammation, we intranasally administered 10 μg LPS 5 times over the course of 10 days starting on day 2 after birth and analyzed the lungs by histology on day 70 (Figure 1A). As expected (14), we observed iBALT structures with separated B and T cell zones and distinct FDC networks in the LPS-treated lungs, but not in the control lungs (Figure 1B). We also quantified the number of GC B cells by flow cytometry and found that GC B cells were present in LPS-treated lungs, but not in control lungs (Figures 1C,D). Given that GCs only form in organized lymphoid tissues, we conclude that pulmonary exposure of neonates to LPS promotes the formation of functional iBALT areas.

To test the effect of iBALT on the immune response to a pulmonary allergen, we administered LPS (or PBS) to neonatal mice as described above, allowed the mice to rest until they were 7 weeks old, then intranasally sensitized the iBALT and control groups with 100 μg OVA in combination with low dose (0.1 μg) LPS on days 49, 50, and 51 and challenged them on days 63, 64, 67, and 68 with 25 μg OVA (Figure 1E). We first examined the GC B cell response in the lungs of sensitized and challenged mice and found that GC B cells were abundant in the lungs of mice with iBALT, but were rare in control mice and almost undetectable in completely naïve mice (Figures 1F,G). We also examined the inflammatory response in the lung and airways by cytospin and found that the proportion (Figures 1H,I) and number (Figures 1J,K) of eosinophils were elevated in mice that had been sensitized and challenged with OVA compared to those in naïve mice, however, the proportion (Figures 1H,I) and number (Figures 1J,K) of eosinophils in mice with iBALT were reduced compared to those in control mice. Reductions in eosinophils were observed in both the lungs (Figures 1H,J) and the airways (Figures 1I,K) of mice with iBALT compared to those in control mice. The proportions of macrophage/monocytes, lymphocytes, and neutrophils are also shown in Figure 1H and the numbers of those cells are shown in Supplementary Figure 1. We also found that total serum IgE concentration was elevated in control mice, but less IgE was detected in mice with iBALT (Figure 1L). Upon a histological examination of lung sections, we found that OVA-exposed mice with pre-existing iBALT had densely-packed areas of lymphocytes to one side of the bronchi, but relatively clear airspaces, whereas OVA-exposed control mice had extensive areas of diffuse inflammation that surrounded the bronchi and extended into the alveolar airspaces (Figure 1M). Thus, contrary to our expectations, the presence of iBALT did not exacerbate OVA-induced pulmonary inflammation, but rather reduced the inflammatory response.

### Th2 Cells Are Generated in Mice With and Without iBALT

**Figure 2:**
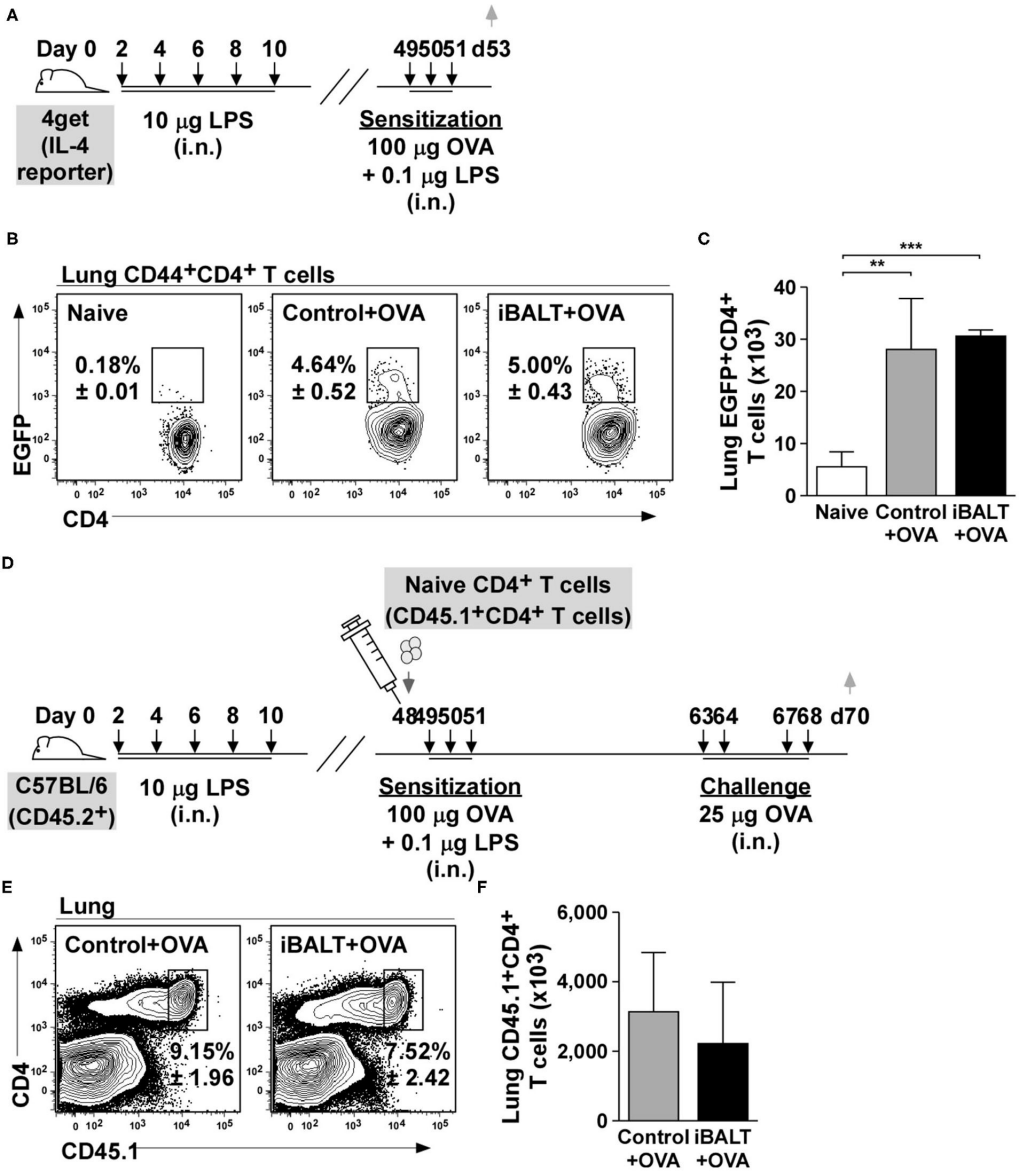
The presence of iBALT does not affect Th2 priming or accumulation in the lung. **(A)** Timing of iBALT induction, allergic sensitization and analysis. **(B,C)** The frequencies **(B)** and numbers **(C)** of EGFP^+^CD4^+^ T cells were determined by flow cytometry. **(D)** Timing of iBALT induction, transfer of naïve OTII cells, allergic sensitization, antigen challenge and analysis. **(E,F)** The frequency **(E)** and number **(F)** of donor T cells in the lungs were determined by flow cytometry. All data show mean ± SD of 5 mice per group; **P < 0.01, ***P < 0.001. Experiments were performed 2 times **(A–C)** or 3 times **(D–F)**.

The presence of iBALT could have altered T cell priming so that fewer Th2 cells were initially generated after antigen exposure. To test this possibility, we next used IL-4 reporter (4get) mice to enumerate Th2 cells. The 4get mice have the enhanced green fluorescent protein (EGFP) targeted into the IL-4 locus with an internal ribosome entry site (IRES) separating the IL-4 coding sequence from the EGFP coding sequence (33). Thus, any cells that express IL-4 mRNA will also express EGFP and can be easily identified using flow cytometry (34). Therefore, we treated neonatal 4get mice with LPS to initiate iBALT formation, waited until they were adults and then intranasally sensitized iBALT and control mice with 100 μg OVA and 0.1 μg LPS on days 49, 50, and 51 (Figure 2A). Two days after the last sensitization, we enumerated IL-4 reporter (EGFP)-expressing CD4^+^ T cells. We found a significant expansion of EGFP^+^CD4^+^ T cells in both iBALT and control mice relative to naïve mice, but did not observe any difference in the frequencies (Figure 2B) or numbers (Figure 2C) of EGFP^+^CD4^+^ T cells between control and iBALT groups. These data suggested that iBALT did not dramatically affect Th2 priming in the lung.

Although the previous assay evaluated Th2 priming, we had no way of enumerating antigen-specific cells. Therefore, to test this possibility in another way, we transferred 1 x 10^6^ naïve OVA-specific CD4^+^ T cells (OT-II cells) into control and iBALT mice on day 48 after birth, sensitized the recipient mice with OVA on days 49, 50, and 51, challenged them on days 63, 64, 67, and 68 and enumerated the responding cells 2 days later in the lung (Figure 2D). We found that the frequency (Figure 2E) and number (Figure 2F) of OT-II cells that accumulated in the lungs of sensitized and challenged mice were the same in control and iBALT groups. These data suggest that the presence of iBALT does not affect OVA-specific T cell accumulation in the lung after sensitization and challenge.

**Figure 3:**
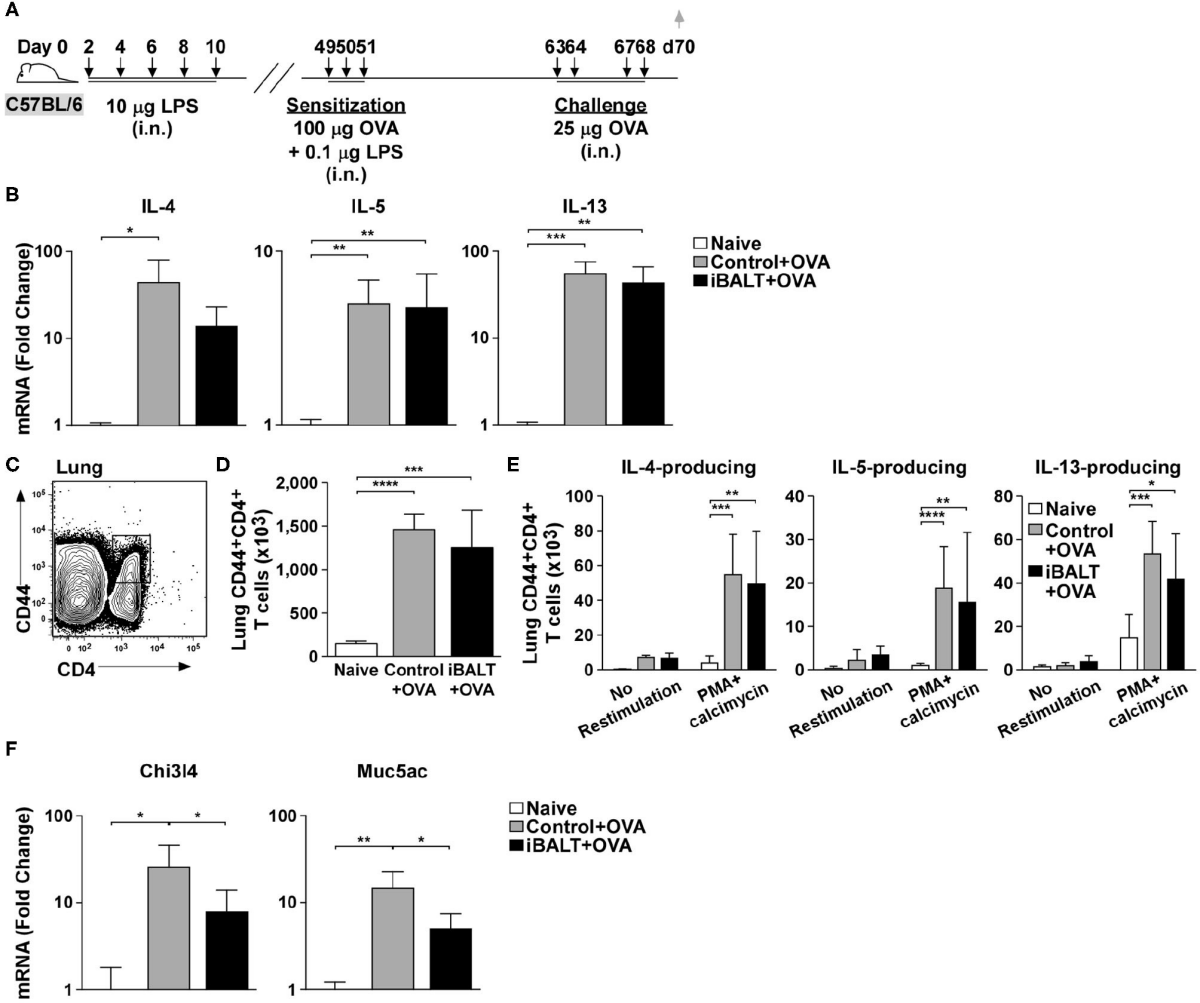
The presence of iBALT impacts allergic airway disease downstream of Th2 cytokine expression. **(A)** Timing of iBALT induction, allergic sensitization, antigen challenge and tissue collection. **(B)** mRNA expression of IL−4, IL−5, and IL−13 was determined by quantitative PCR. Data is normalized to the expression in naïve lung. **(C–E)** Lung cells from OVA primed and challenged mice were restimulated with PMA/calcimycin for 4 h and we determined the frequencies **(C)** and numbers **(D)** of CD4^+^CD44^hi^ T cells as well as the numbers of cytokine–producing CD4^+^ T cells **(E)** by intracellular staining and flow cytometry. **(F)** mRNA expression of Chi3l4 and Muc5ac was determined by quantitative PCR. Data is normalized to the expression in naïve lung. All data show mean ± SD of 5 mice per group; *P < 0.05, **P < 0.01, ***P < 0.001, ****P < 0.0001. Experiments were performed 3 times **(B,F)** or 4 times **(C–E)**.

Although Th2 priming and expansion appeared normal in mice with iBALT, it was not clear whether the final effector cells could actually make Th2 cytokines. Therefore, to determine whether CD4 T cells primed by OVA sensitization and challenge could actually produce Th2 cytokines, we sensitized and challenged mice with and without pre-existing iBALT and measured the mRNA expression of Th2 cytokines in the lungs 48 h after the last OVA challenge (Figure 3A). We found that mRNAs encoding IL-4, IL-5, and IL-13 were increased in OVA-exposed and challenged lungs relative to their expression in naïve lungs, but we did not observe a significant difference between the control and iBALT groups (Figure 3B).

We next tested whether CD4^+^ T cells from OVA sensitized and challenged control and iBALT mice were equally capable of making Th2 cytokines. To test this possibility, we collected cells from the lung tissue, restimulated them with PMA and calcimycin for 4 h and assessed the production of IL-4, IL-5, and IL-13 by intracellular staining. We found that the total number of activated CD44^hi^CD4^+^ T cells was increased in control and iBALT mice relative to that in naïve mice (Figures 3C,D), but there was little difference between the control and iBALT groups. We also found that the numbers of CD44^hi^CD4^+^ T cells that made IL-4, IL-5, or IL-13 were increased in control and iBALT mice relative to those in naïve mice (Figure 3E), but there was little difference between the control and iBALT groups. These data suggest that the presence of iBALT does not significantly alter the number of T cells capable of producing Th2 cytokines following sensitization and challenge.

Th2 cytokines act on other cells in the lung, such as epithelial cells and macrophages, and promote their activation and differentiation, which changes gene expression. For example, genes like chitinase-3-like-4 (Chi3l4) and mucin 5ac (Muc5ac) are expressed in in the lung following IL-13 signaling (35). Thus, we next examined the expression of mRNAs encoding these genes in the lungs of iBALT and control mice after OVA sensitization and challenge as in Figure 3A. We found that mRNAs encoding Chi3l4 and Muc5ac were increased in OVA-exposed and challenged lungs relative to their expression in naïve lungs, but their expression in the iBALT group was significantly less than that in the control group (Figure 3F). Thus, the expression of genes that are responsive to Th2 cytokines were reduced in the lungs of iBALT mice relative to those in control mice. These data are more consistent with the reduced histopathology that we see in the lungs of iBALT mice compared to those in the lungs of control mice, suggesting that the difference between the groups is not in Th2 priming and expansion, but in the response to Th2 cytokines.

### The Presence of iBALT Alters Pulmonary Pathology Independently of Th2 Priming

**Figure 4:**
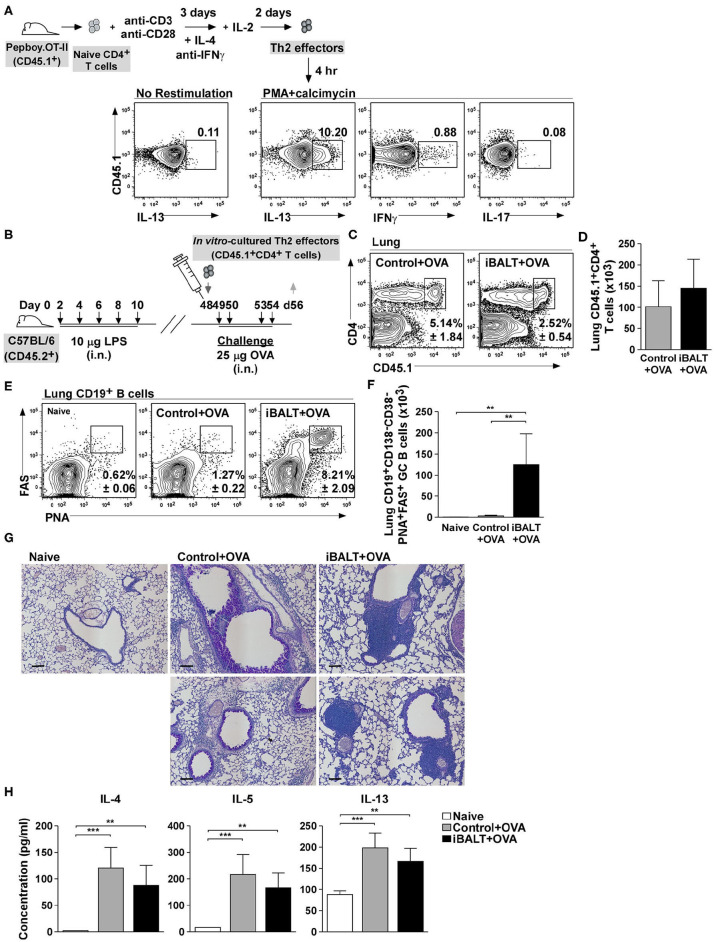
iBALT alters pulmonary pathology independently of Th2 effector priming. **(A)** Purified CD4^+^ OTII cells were expanded *in vitro* under Th2 conditions and their expression of IL−13, IFNγ, and IL−17 was determined by intracellular staining and flow cytometry after 4 h restimulation with PMA and calcimycin. **(B)** *In vitro*–activated Th2 cells were adoptively transferred into iBALT or control mice on day 48 and the recipients were intranasally challenged with OVA on days 49, 50, 53, and 54. **(C,D)** The frequencies **(C)** and numbers **(D)** CD45.1^+^CD4^+^ T cells in the lungs were determined by flow cytometry on day 56. **(E,F)** The frequencies **(E)** and numbers **(F)** of CD19^+^CD138–CD38–PNA^+^FAS^+^ GC B cells in the lung were determined by flow cytometry. **(G)** Periodic–acid schiff (PAS) staining of paraffin–embedded lung sections on day 52 (scale bar = 100 μm). **(H)** The concentration of IL−4, IL−5, and IL−13 in the BAL fluid was determined by ELISA 6 h after the last OVA challenge. All data show mean ± SD of 4–5 mice per group; **P < 0.01, ***P < 0.001. Experiments were performed 5 times **(A–G)** or 2 times **(H)**.

In the experiments above, we were unable to independently control the initial steps of T cell priming and expansion. To rigorously show that these steps were unaffected by the presence or absence of iBALT, we next generated OVA-specific Th2 effector T cells *in vitro* and showed that, upon restimulation, a high frequency of these cells made IL-13, whereas less than 1% made IFNγ and almost none of them made IL-17 (Figure 4A). We next adoptively transferred 1 x 10^6^ of these Th2 cells into 48-day-old control or iBALT mice and challenged them with OVA on days 49, 50, 53, and 54 (Figure 4B). One day after the final challenge, we enumerated donor T cells in the lungs and found that a similar frequency (Figure 4C) and number (Figure 4D) of donor T cells had accumulated in the lungs of both groups. We also enumerated GC B cells in the lungs and found that a high frequency (Figure 4E) and number (Figure 4F) of GC B cells accumulated in the lungs of mice in the iBALT group, but not in the lungs of control or naïve mice.

Despite having similar numbers of donor Th2 cells in the lungs of each group, the histology of the lungs was dramatically different, with the control group exhibiting extensive goblet cell hyperplasia and diffuse inflammatory infiltrate throughout the lungs, whereas the iBALT mice exhibited minimal goblet cell hyperplasia and dense lymphoid accumulations (iBALT) adjacent to, but not surrounding, the airways and in the perivascular space (Figure 4G). We also quantified the amount of Th2 cytokines in the BAL fluid 6 h after the last challenge and found that, although the amount of IL-4, IL-5, and IL-13 was slightly less than in the BAL fluid of iBALT mice than in control mice, the differences were not significant (Figure 4H). Together, these data demonstrate that, despite starting with the same number of Th2 cells in the lung, the biological outcome of inflammation was dramatically different in control and iBALT mice.

### The Presence of iBALT Does Not Alter Treg Accumulation or Epithelial Cell Conditioning

**Figure 5:**
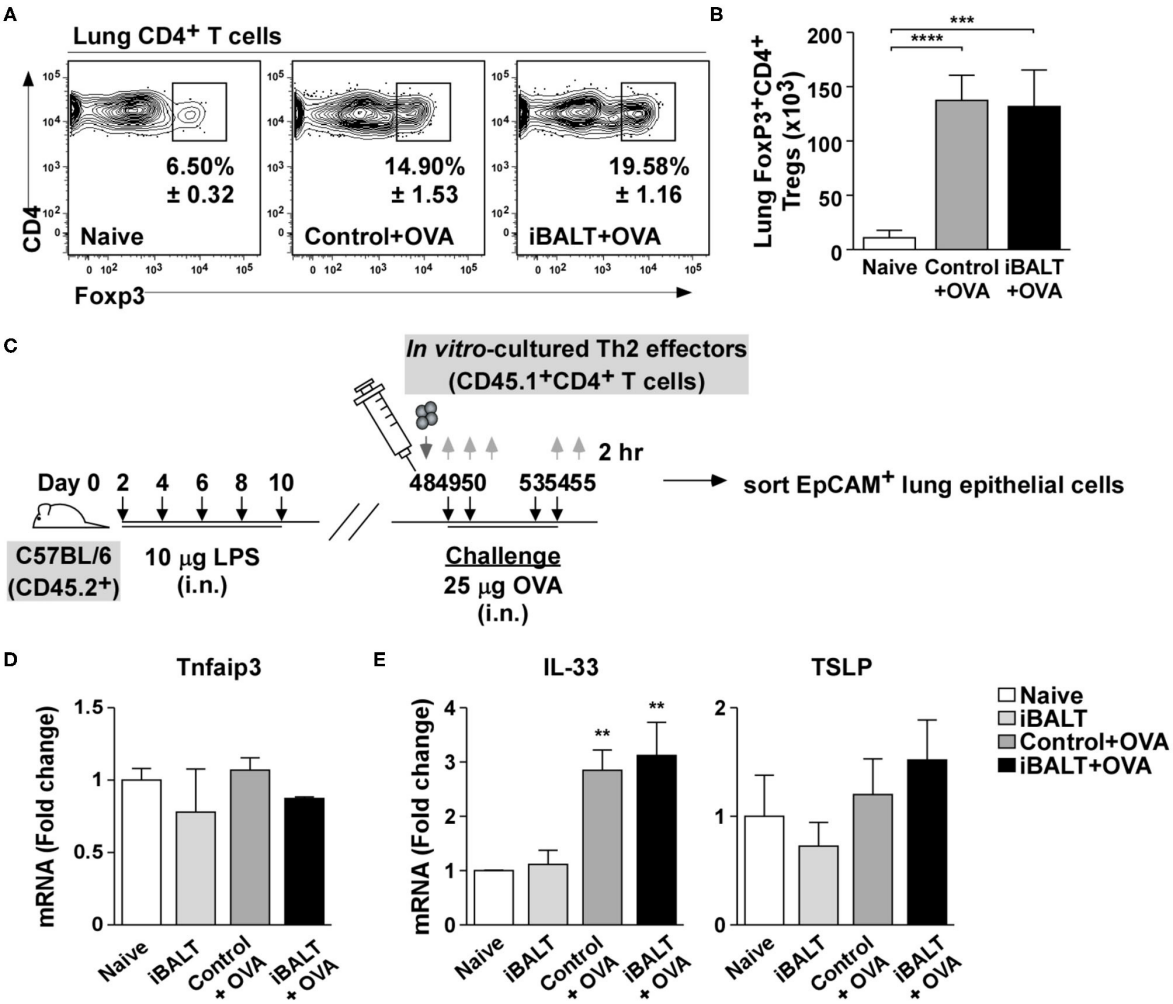
The presence of iBALT does not influence Treg accumulation or epithelial cell conditioning. Mice with and without iBALT were sensitized and challenged with OVA according to the schedule in Figure 1E and the frequencies **(A)** and numbers **(B)** of Foxp3^+^CD4^+^ T cells were determined by flow cytometry (mean ± SD of 4–5 mice per group; ***P < 0.001, ****P < 0.0001). **(C)** Timing of iBALT induction, Th2 transfer, antigen challenge and tissue collection. **(D,E)** mRNA expression of **(D)** Tnfaip3 and **(E)** IL-33 and TSLP from sorted lung epithelial cells was determined by quantitative PCR 2 h after the last OVA challenge. Data is normalized to the expression in naïve lung (mean ± SD of 4–5 mice pooled per group; **P < 0.01). Experiments were performed 4 times **(A,B)** or 3 times **(C–E)**.

Given that the biological outcome of disease was so different despite starting with similar numbers of fully differentiated Th2 cells, we reasoned that iBALT must engage some sort of regulatory mechanism. One important cell type that has the ability to powerfully attenuate inflammatory responses is the Treg population (36). Tregs are immunosuppressive CD4^+^ T cells that have differentiated in a way that promotes the expression of the transcription factor, FoxP3 (37). CD4^+^FoxP3^+^ Tregs can reduce eosinophilia (38), impair humoral responses and secret an inhibitory cytokines, such as IL-10 and TGFβ (39), which suppress the effector functions of activated T cells and reduce inflammation. Thus, changes in Treg number or activity can have dramatic consequences on immune responses regardless of the number of antigen-specific effector T cells. Given the immunosuppressive capacity of Tregs, we enumerated CD4^+^FoxP3^+^ T cells in the lungs of iBALT and control mice following OVA sensitization and challenge. We found that although the frequency (Figure 5A) and number (Figure 5B) of Tregs increased in the lungs following OVA sensitization and challenge, there was no difference in Tregs between iBALT and control groups. Thus, alterations in Treg numbers do not explain why mice with iBALT exhibit attenuated allergic responses in their lungs.

Previous reports show that pulmonary administration of LPS to the lung conditions epithelial cells in a way that promotes the over-expression of A20 (40), an ubiquitin-modifying enzyme that blunts NF-kB signaling thereby reducing expression of pro-Th2 cytokines like IL-33 (41). Thus, we were concerned that the exposure of neonatal mice to LPS may permanently suppress pulmonary inflammatory responses. To test this possibility, we treated neonatal mice with LPS or PBS to trigger iBALT formation and, when the mice were 7-weeks old, transferred OVA-specific Th2 effector cells and challenged the recipients 4 times with either OVA or PBS (Figure 5C). We then enzymatically digested the lung tissue, sorted EpCAM^+^CD31^−^Sca1^−^CD45^−^ bronchial epithelial cells and performed qPCR for *Tnfaip3*, the gene encoding the A20 enzyme. We found that *Tnfaip3* expression was similar in all groups of bronchial epithelial cells (Figure 5D), regardless of whether they came from mice that received LPS as neonates or whether they came from mice that were challenged with OVA as adults. We also performed qPCR to quantify the expression of the inflammatory cytokines, IL-33 and TSLP. We found that although IL-33 expression was strongly increased following OVA challenge, it was not altered by previous exposure to LPS (Figure 5E). We also found that TSLP expression was not changed by either OVA challenge or previous exposure to LPS (Figure 5E). Thus, the pulmonary exposure of neonatal mice to LPS did not permanently alter the ability of bronchial epithelial cells to express inflammatory cytokines.

### Altered Kinetics of Effector Th2 Responses in the Lungs of Mice With iBALT

**Figure 6:**
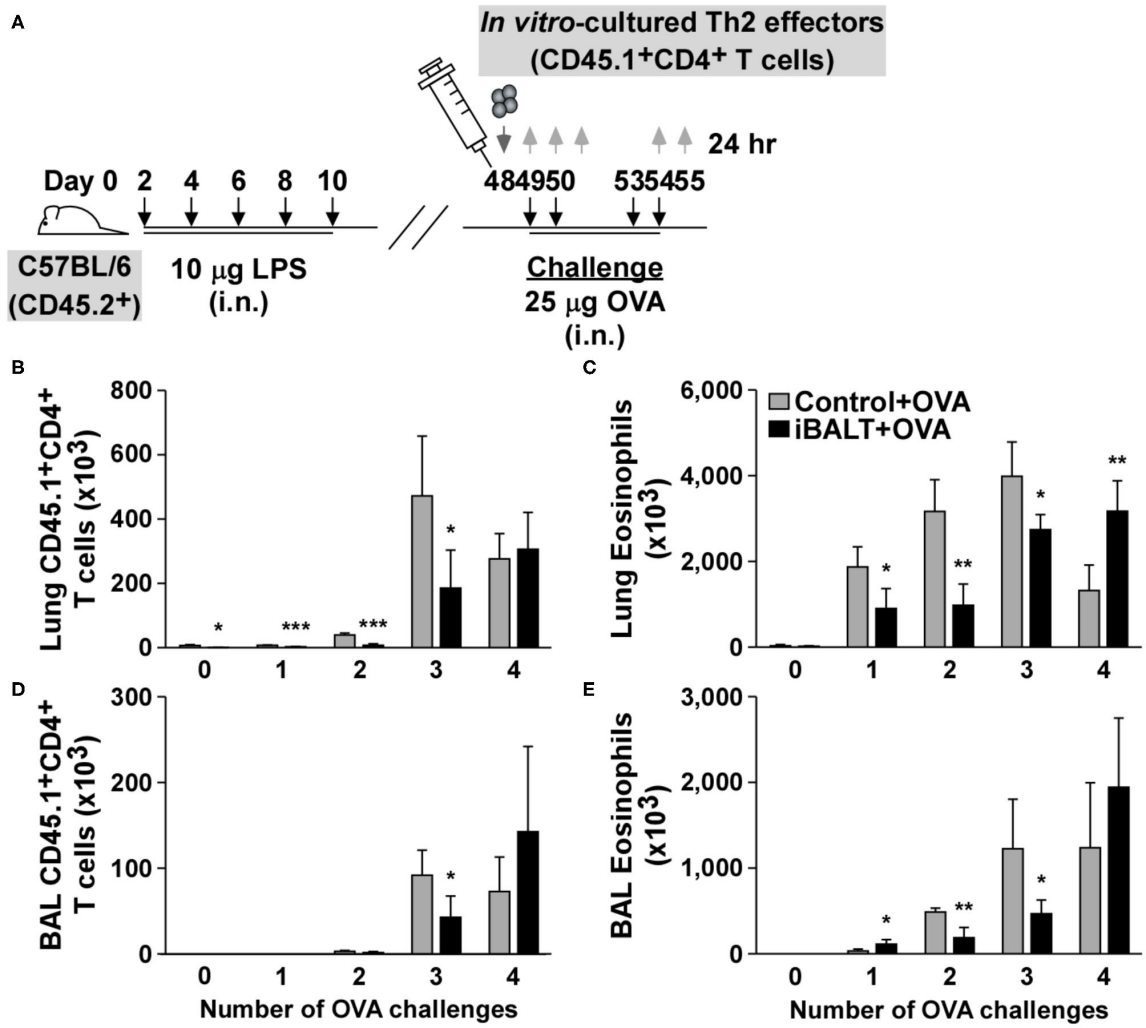
The presence of iBALT alters the kinetics of Th2 accumulation in the lungs. **(A)** Timing of iBALT induction, Th2 effector cell transfer and antigen challenge. Tissues were collected 24 h after each challenge. The numbers of donor T cells **(B)** and eosinophils **(C)** in the lung were determined by flow cytometry. The numbers of donor T cells **(D)** and eosinophils **(E)** in the BAL fluid were determined by flow cytometry. All data show mean ± SD of 4–5 mice per group; *P < 0.05, **P < 0.01, ***P < 0.001. Experiments were performed 3 times **(A–E)**.

Despite the overall reduction in Th2-driven pathology in the mice with iBALT, the number of Th2 effector cells seemed to be similar at the endpoint of the experiment. However, we didn't know whether they accumulated in the lung at the same rate. To determine whether the kinetics of T cell recruitment was the same in mice with and without iBALT, we generated Th2 effectors from naïve OTII cells *in vitro* and then adoptively transferred 1 x 10^6^ Th2 effectors 24 h prior to a series of 4 OVA challenges (Figure 6A). We then enumerated the transferred Th2 cells and the recruited eosinophils in the lungs and BAL 24 h after each OVA challenge. Not surprisingly, the numbers of donor T cells and eosinophils increased over time following each challenge (Figures 6B–E). Unexpectedly however, we observed significant reductions in the numbers of Th2 effectors (Figures 6B,D) and eosinophils (Figures 6C,E) in both the lungs (Figures 6B,C) and airways (Figures 6D,E) of iBALT mice relative to controls after the first 3 challenges. However, by the fourth challenge, there was no difference in the numbers of T cells. These data suggest that the presence of iBALT slows the accumulation of Th2 effectors as well as eosinophils following pulmonary allergen exposure. These data may also explain why we saw little difference in T cell numbers at the endpoints of earlier experiments, even though we observed significant differences in lung pathology.

### The Presence of iBALT Alters the Positioning of Th2 Cells in the Lung

**Figure 7:**
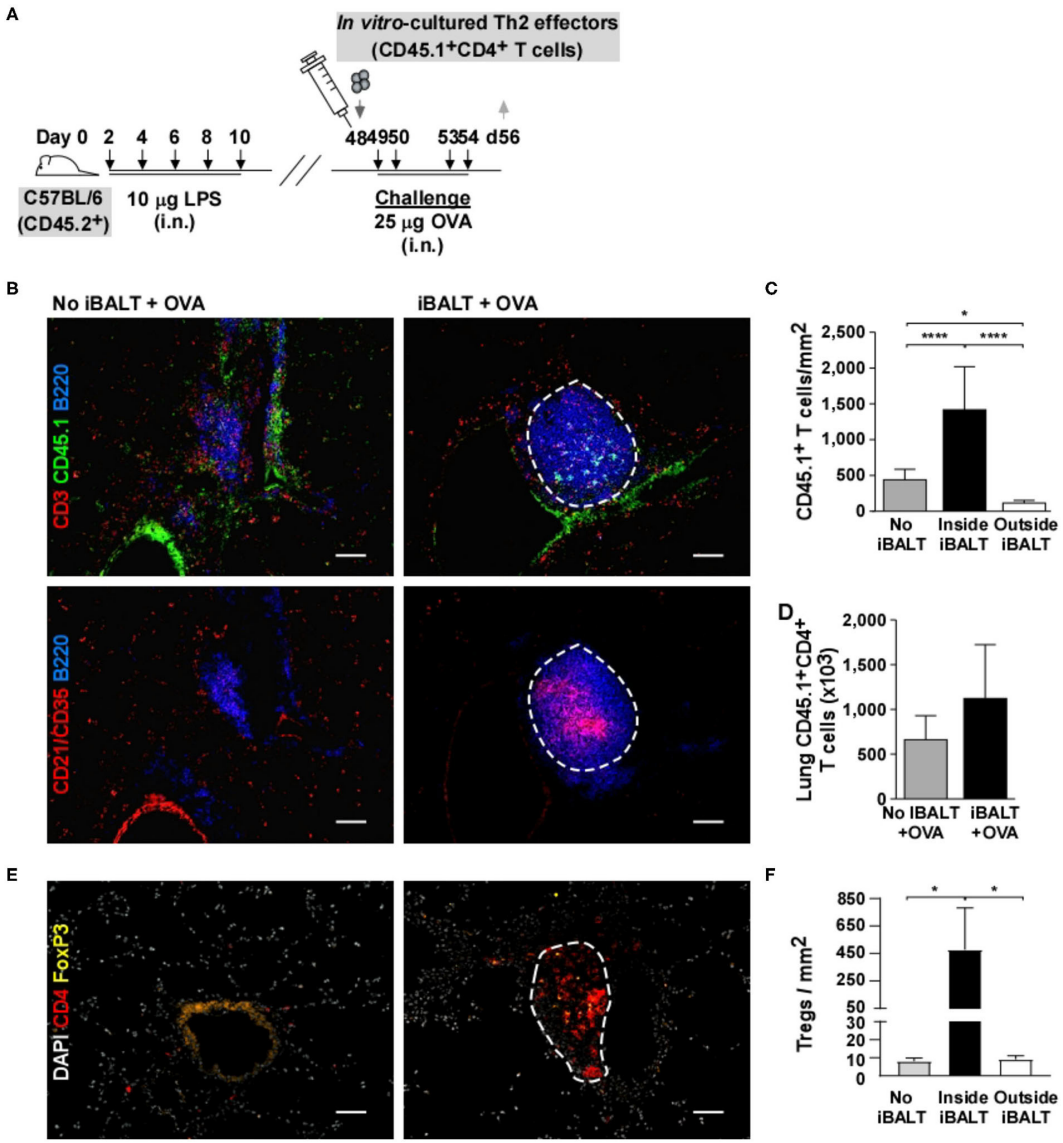
The presence of iBALT alters the spatial distribution of T cells. **(A)** Timing of iBALT induction, effector Th2 transfer and antigen challenge. **(B)** Serial cryosections were probed with antibodies against CD3, CD45.1 and B220 or with antibodies against CD21/CD35 and B220 as indicated. Images were obtained with 20X objective (scale bar = 100 μm). **(C)** The frequency of CD45.1^+^ T cells per μm^2^ was determined in areas of iBALT (an example is outlined in white) or in non–iBALT areas using a mosaic of high–magnification images. **(D)** The numbers of lung CD45.1^+^CD4^+^ T cells were determined by flow cytometry. **(E)** Cryosections were probed with antibodies against CD4 and FOXP3. Images were obtained with 20X objective (scale bar = 100 μm). **(F)** The frequency of CD45.1^+^ T cells per μm^2^ was determined in areas of iBALT (an example is outlined in white) or in non–iBALT areas using a mosaic of high–magnification images. All data show mean ± SD of 4–5 mice per group; *P < 0.05, ****P < 0.0001. Experiments were performed 3 times **(A–D)** or 1 time **(E,F)**.

The most unique attribute of iBALT is its dense accumulations of spatially ordered lymphocytes in a tissue (the lung) that normally has relatively few lymphocytes and rarely has them so densely organized. Therefore, we next asked whether the specialized environment of iBALT alters the spatial organization of T cells in the lung following Ag exposure. To test this possibility, we generated OVA-specific Th2 effector cells *in vitro* using OTII cells. These cells were subsequently transferred into iBALT and control mice and the recipients were challenged with OVA 4 times before we examined the lungs by histology (Figure 7A). We performed immunofluorescence looking for the donor T cell marker (CD45.1), B cells (B220), and FDCs (CD21). We found that the donor effector Th2 cells preferentially accumulated in the iBALT areas of iBALT mice and were relatively dilute in the rest of the lung (Figure 7B). In contrast, the donor Th2 effector cells were distributed more evenly in the control mice. These data were quantified in Figure 7C. Importantly, we found that the total number of donor Th2 effector cells in the lungs of mice with and without iBALT were indistinguishable at this time (Figure 7D). We also examined CD4+FOXP3+ Tregs in tissue sections from mice with and without iBALT. We found that Tregs were densely clustered in areas of iBALT, but were relatively dilute in the rest of the lung (Figures 7E,F), despite the similar numbers of Tregs in the lungs of mice with and without iBALT (Figures 5A,B). Together, these data suggest that the spatial distribution of effector Th2 cells and Tregs is affected by the presence of iBALT (they cluster together), which may explain how iBALT and control mice can have similar numbers of Th2 cells in their lungs, but have so profoundly different outcomes in terms of eosinophil accumulation and histopathology.

### Pulmonary Exposure, but Not Systemic Exposure, to LPS Promotes iBALT and Reduces Allergen-Induced Inflammation

**Figure 8:**
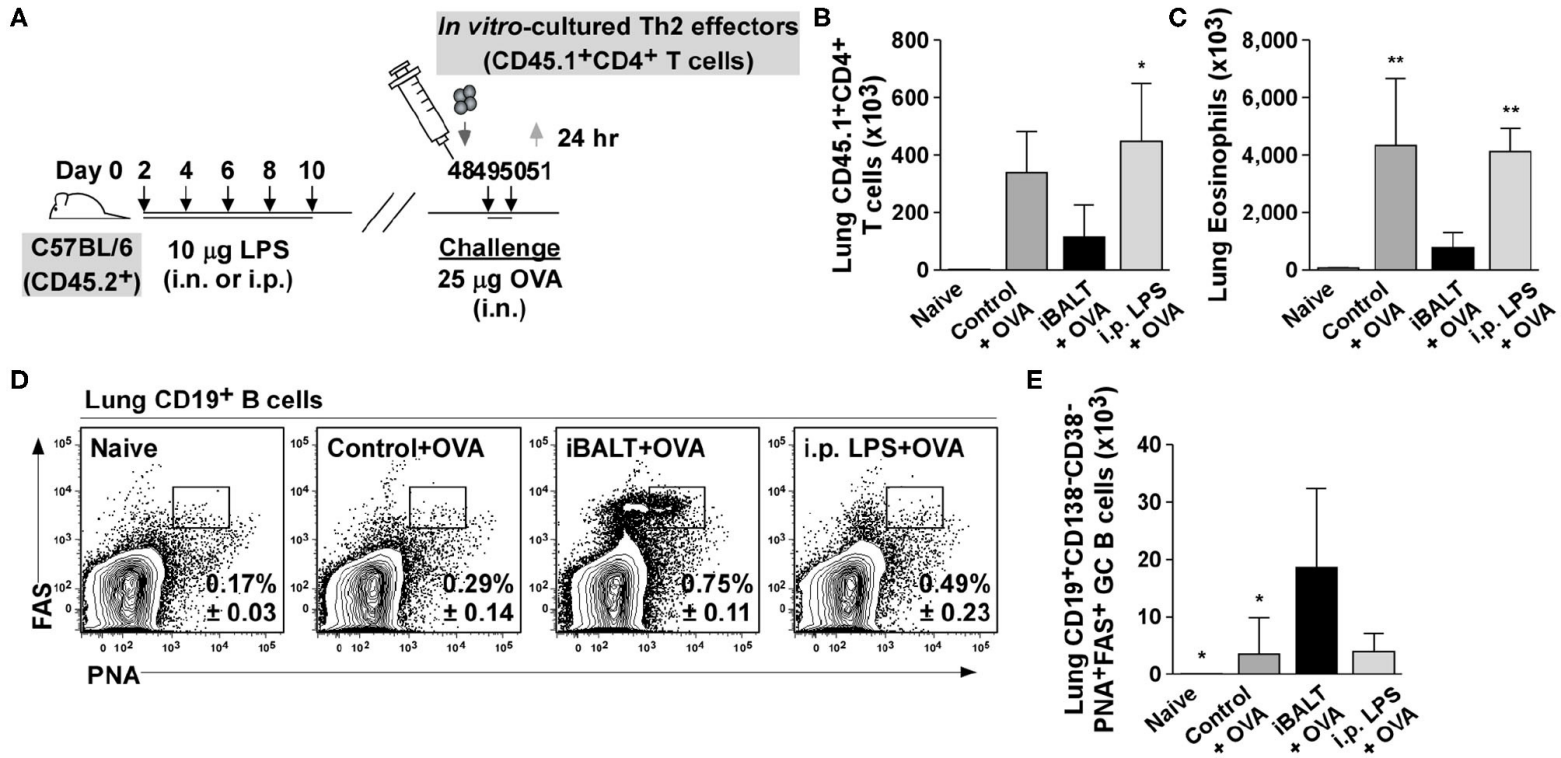
Pulmonary, but not systemic LPS administration leads to iBALT formation and reduced inflammatory responses in the lung. **(A)** Timing of iBALT induction, transfer of effector Th2 cells and antigen challenge. The numbers of donor T cells **(B)** and eosinophils **(C)** in the lungs was demined by flow cytometry 24 h after the last OVA challenge. The frequency **(D)** and number **(E)** of GC B cells in the lungs was determined 24 h after the last OVA challenge by flow cytometry. All data show mean ± SD of 4–5 mice per group; *P < 0.05, **P < 0.01. Experiments were performed 3 times **(A–E)**.

The above experiments used mice that had been administered LPS as neonates in order to form iBALT. However, one concern with these experiments is that neonatal exposure to LPS might have altered systemic immunity in some way that ameliorated subsequent inflammatory responses independently of iBALT formation. To rule out this possibility, we generated *in vitro*–differentiated OTII Th2 cells and transferred 1 x 10^6^ Th2 effector cells into control mice, mice that received pulmonary LPS as neonates (iBALT mice) and mice that received peritoneal LPS as neonates (i.p. LPS mice). The following 2 days, we administered 25 μg OVA intranasally to all groups and measured germinal center B cells and eosinophils in the lungs 24 h later (Figure 8A).

When we enumerated Th2 effectors (Figure 8B) and eosinophils (Figure 8C) in the lung tissue, the numbers were reduced in the presence of iBALT compared to control mice, but in mice treated with LPS intraperitoneally, the number of effector T cells and eosinophils were the same as in the control mice (Figures 8B,C). Thus, the exposure of neonates to pulmonary LPS dramatically decreased the inflammatory response in adults, whereas the exposure of neonates to peritoneal LPS did not. We also observed that mice that receive pulmonary LPS as neonates (iBALT mice) had a significantly higher frequency (Figure 8D) and number (Figure 8E) of GC B cells in the lungs, whereas mice that received LPS intraperitoneally as neonates (i.p. LPS mice) did not accumulate GC B cells and were comparable to control mice (Figures 8D,E). These data indicate that systemic exposure to LPS does not lead to iBALT formation (as monitored by the germinal center response in the lungs). These data are consistent with the conclusion that LPS-mediated iBALT formation alleviates Th2-dependent inflammatory responses in the lung, independently of any systemic effect of LPS on immunity.

## Discussion

Our data show that the presence of iBALT in the lungs prior to pulmonary sensitization and challenge with OVA does not exacerbate Th2 responses, but rather attenuates Th2-driven pulmonary pathology. In fact, mice with iBALT exhibit delayed Th2 accumulation, reduced eosinophil recruitment, reduced goblet cell hyperplasia and reduced mucus production compared to their control counterparts. Although the initial priming of Th2 cells is not affected by the presence of iBALT, the accumulation of Th2 cells in the lung is delayed following pulmonary challenge. More importantly, the spatial distribution of effector T cells is altered by the presence of iBALT, such that effector CD4 T cells as well as Tregs become concentrated in iBALT areas and relatively diluted in the rest of the lung. These results suggest that iBALT functionally sequesters effector T cells, thereby limiting the exposure of the lung parenchyma to T cell-produced inflammatory cytokines, which attenuates pulmonary inflammation and prevents excessive pathology.

IBALT is associated with a wide variety of inflammatory lung diseases, including COPD (26), hypersensitivity pneumonitis (27) and rheumatoid lung disease (3), all of which are the result of chronic exposure to antigens or inflammatory agents. Although asthma is also a chronic lung disease, the mouse model of repeated sensitization and challenge with OVA is more like an acute allergic response. However, our data demonstrate that the mere presence of iBALT does not necessarily lead to an increased inflammatory response or pulmonary pathology in the context of pulmonary allergen exposure as one might expect if iBALT was facilitating antigen presentation and T cell activation. Therefore, despite participating in local immune responses, iBALT may be beneficial in the context of inflammatory diseases by sequestering activated lymphocytes. The idea of reducing inflammation by sequestering T cells in lymphoid tissue is consistent with the activities of the immunosuppressant drug, FTY720, which prevents T cells from exiting lymphoid organs—effectively sequestering them and preventing their recirculation through peripheral tissues (42, 43). In fact, treatment with FTY720 reduces airway remodeling and pulmonary inflammation in a rat model of OVA-induced asthma (44). Interestingly, iBALT areas are expanded in the lungs of FTY720-treated animals, suggesting that the FTY720-mediated sequestration of lymphocytes can occur in either conventional lymphoid organs or in areas of iBALT in the lung (13).

Another physical change that occurs in the lung concomitantly with the development of iBALT is the formation of additional lymphatic vessels surrounding the iBALT follicles (45). Live imaging of iBALT suggests that these lymphatic vessels may facilitate the collection of DCs from the airways (46). Static imaging also shows that iBALT areas collect inhaled antigens and particulates (47), suggesting that iBALT may sequester antigen as well as T cells. In this context, it is interesting to note that mice that spontaneously develop iBALT in the context of rheumatoid lung disease are highly resistant to developing fibrosis following pulmonary challenge with bleomycin (48). If iBALT areas efficiently collect and sequester intratracheally administered bleomycin or remove it from the lung via lymphatics, then one would expect to observe reduced fibrosis. Similarly, if iBALT areas collect and sequester pulmonary antigens like OVA and remove them from the lung parenchyma, one would expect reduced pulmonary inflammation. Thus, iBALT may protect the lung by collecting antigens and inflammatory substances as well as T cells.

Our data are also consistent with observations that iBALT attenuates inflammatory responses and improves clinical outcomes following infection with a variety of pulmonary pathogens. For example, the presence of iBALT promotes the survival of mice that are challenged with normally lethal doses of influenza virus, pneumovirus and SARS corona virus (24) and attenuates the pulmonary pathology associated with these infections. Much of the clinical pathology, such as weight loss, associated with pulmonary viral infections is linked to the production of inflammatory cytokines by T cells. Thus, if iBALT sequesters virus-specific effector T cells, it may limit the exposure of the rest of the lung to inflammatory cytokines and chemokines and reduce the recruitment of additional inflammatory cells—thereby attenuating pathology. However, iBALT-mediated resistance to pneumovirus is associated with impressive increases, rather than decreases, in the mRNA expression of IFNγ, CXCL10, and CCL3 in the lung (12). These responses were measured by PCR using mRNA extracted from whole lungs, so it is difficult to know whether the increases were predominantly confined to iBALT areas or not. In addition, it is not clear whether the reported increase is due to an overall increased magnitude of the response or altered kinetics, such that more T cells are responding earlier. This last possibility seems plausible, given that accelerated antibody responses are also observed in mice with iBALT (24). Interestingly, we also observe altered kinetics of effector T cell recruitment to the lungs following pulmonary challenge with allergen. However, we find that fewer effector Th2 cells accumulate in the lungs at early times after allergen exposure. The differences between our results and those reported for pneumovirus may be due to the types of antigens (replicating virus vs. inert allergen) or to the types of T cells (virus–specific CD8 and Th1 cells vs. allergen-specific Th2 cells). In any case, the presence of iBALT clearly has an impact on the kinetics of pulmonary immune responses.

T cells are often imprinted with particular effector functions that differ depending on the secondary lymphoid organ in which they were primed (49, 50). Thus, one could envision that iBALT skews CD4 T cell differentiation away from a Th2 pathway and thereby attenuates the symptoms of allergic disease. However, we found equivalent numbers and frequencies of Th2 cells primed in mice with or without iBALT, suggesting that CD4 T cell differentiation is not deviated toward an alternative effector function. Moreover, we observed reduced pathology in mice with iBALT following the adoptive transfer of *in vitro* differentiated Th2 effector cells, demonstrating that even when T cell priming occurs *in vitro*, the functional outcome of the effector response is altered beneficially in mice with iBALT. Interestingly, naïve T cells primed in aorta–associated lymphoid tissues in Apoe^−−/−−^ mice preferentially differentiate into Tregs that suppress atherosclerosis (51). Although the preferential formation of Tregs in iBALT areas would help to explain the reduced inflammation and pulmonary pathology in mice with iBALT following OVA sensitization and challenge, we did not observe changes in either the number and frequency of Tregs in the lung, regardless of whether iBALT was present. Nevertheless, we did find that Tregs were preferentially concentrated in iBALT areas and co-localized with effector T cells, suggesting that Tregs in iBALT may more effectively suppress the inflammatory functions of effector T cells in this location. Thus, in the context of pulmonary sensitization with allergens such as OVA, iBALT seems to alter the placement of T cells rather than changing their differentiation pathways.

Considering that many of the adoptively transferred Th2 cells are being sequestered in the iBALT areas and seem to be located in the B cell follicle, it is possible that they are converting to IL-4-expressing Tfh cells (52). This possibility would be consistent with the large GCs and high frequencies of GC B cells that we observe in the lungs following adoptive transfer of Th2 cells and sensitization with antigen. In addition, iBALT-induced resistence to influenza and SARS corona virus is associated with accelerated antibody responses (24), suggesting that local Tfh cells are promoting B cell differentiation. Although Th2-Tfh cells in B cell follicles are potent producers of IL-4 (53), they are directing cytokine secretion toward B cells rather than pulmonary epithelial cells, which would likely lead to accelerated antibody secretion, but reduced pulmonary pathology. Thus, iBALT may be sequestering T cells and directing cytokine secretion toward alternative cell types, both of which should attenuate pulmonary pathology.

In summary, our data show that the presence of iBALT does not exacerbate pulmonary pathology following sensitization and challenge with an allegen, but rather attenuates Th2-induced inflammatory responses. This same mechanism may also explain the ability of iBALT to ameliorate pulmonary pathology in the context of infection and may be a general mechanism by which ectopic follicles in a variety of tissues attempt to cope with chronic inflammatory responses. Thus, we may want to develop treatments that promote, rather than prevent, ectopic follicle formation in the context of chronic inflammatory conditions.

## Materials and Methods

### Mice

C57BL/6, B6.SJL–Ptprc^a^Pepc^b^/BoyJ (CD45.1) and B6.Cg–Tg(TcraTcrb)425Cbn/J (OTII) mice were originally obtained from Jackson Laboratories. These strains were interbred to generate CD45.1–OTII mice. B6.129–*Il4*^*tm*1*Lky*^/J (4get) mice were obtained from M. Mohrs. All mice were on a C57BL/6 background and were bred at the University of Rochester or University of Alabama at Birmingham animal facilities and all experimental procedures were approved by the University of Rochester University Committee on Animal Resources (UCAR) or University of Alabama at Birmingham Institutional Animal Care and Use Committee (IACUC) and were performed according to guidelines outlined by the National Research Council.

### Induction of iBALT, Ova Sensitization, and Challenge

To induce iBALT, pups were intranasally administered 10 μg LPS from *Escherichia coli* (055:B5; Sigma) in 10 μl PBS every other day starting on day 2 after birth for a total of 5 times. In control experiments, pups were intraperitoneally injected with 10 μg LPS in 10 μl PBS every other day starting on day 2 after birth for a total of 5 times. Control mice received 10 μl PBS or nothing according to the same schedule. To induce allergic airway inflammation, adult mice were sensitized intranasally with 100 μg OVA (Sigma) plus 0.1 μg LPS in 100 μl PBS and subsequently challenged intranasally with 25 μg OVA in 100 μl PBS according to the schedule indicated in each experiment. In the DC labeling experiments, we intratracheally administered 25 μg OVA labeled with Alexa Flour® 647 (Invitrogen).

### OTII Purification, Th2 Differentiation, and Adoptive Transfer

CD4^+^ T cells were purified from the spleens of naive CD45.1^+^ B6 congenics using LS columns and anti–CD4 MACS beads (Miltenyi Biotec) according to the manufacturer's instructions. Single T cell preparations were > 95% pure as determined by flow cytometry. Th2 effectors were generated by activating naïve T cells for 48–72 h with plate–bound anti–CD3 (2 μg/ml; 145–2C11; BioXcell) and anti–CD28 (0.5 μg/ml; 37.51; eBioscience) in the presence of IL−4 (50 U/ml; DNAX) and anti–IFNγ (10 μg/ml; XMG1.2; eBioscience) followed by feeding with IL−2 (20 U/ml; Peprotech) for additional 48 h. Naïve and effector CD4^+^ (CD45.1^+^) T cells (1 x 10^6^/mouse) were injected intravenously into no iBALT and iBALT recipients that were subsequently immunized with OVA.

### Cell Preparation and Flow Cytometry

Lungs were cut into small fragments and digested with 0.6 mg/ml collagenase A (Sigma) and 30 μg/ml DNase I (Sigma) in RPMI 1640 medium (Gibco) at 37°C for 45 min to obtain single–cell suspensions. Digested tissues were mechanically disrupted by passage through a metal strainer. Cell suspensions from lungs and mediastinal LNs were centrifuged and resuspended in 150 mM NH_4_Cl, 10 mM KHCO_3_, and 0.5 mM ethylenediaminetetraacetic acid (EDTA; Lonza) for 5 min to lyse red cells. Cell suspensions were then filtered through a 70 μm nylon cell strainer (BD Biosciences), washed, and resuspended in phosphate–buffered saline (PBS) with 5% donor calf serum and 10 μg/ml Fc Block (2.4G2; Trudeau Institute) on ice for 10 min before staining with fluorochrome–conjugated antibodies against the following antigens: CD4 (RM4–5), CD11b (M1/70), CD25 (PC61), CD45R (B220; RA3–6B2), CD95 (FAS; Jo2), CD138 (281–2), Siglec–F (E50–2440), and I–A^b^ (AF6–120.1; all from BD Biosciences); CD11c (N418), CD44 (IM7), CD45.1 (A20), CD103 (2E7), and Foxp3; (FJK−16s); all from eBioscience; CD19 (6D5); from BioLegend; CD38 (NIMR−5); from SouthernBiotech; and goat anti–rabbit–Alexa Flour® 647 (Invitrogen). For intracellular staining, single–cell suspensions from the lungs were stimulated on plates coated with anti–CD3 (2 μg/ml; 145–2C11; BioXcell) in the presence of Brefeldin A (12.5 μg/ml; Sigma) for 4 h. The restimulated cells were surface stained, then fixed in 4% paraformaldehyde, made permeable with 0.1% Triton™ X−100 (Sigma), and stained with anti–IL−4 (11B11), anti–IL−5 (TRFK4; all from BD Biosciences) or anti–IL−13 (13A; eBioscience). Cells were analyzed with a LSR II (BD Biosciences) or FACSCanto II (BD Biosciences) located at the University of Rochester and University of Alabama at Birmingham Flow Cytometry Core Facility.

To isolate lung epithelial cells, we perfused the lungs with PBS containing 0.05 mM EDTA (Lonza), instilled with 1 ml Dispase (160 μg/ml; Corning), and incubated them in a shaker at 37°C for 15 min. Using forceps, lungs were torn into small pieces, and put back in the shaker at 37°C for an additional 30 min in Dispase (160 μg/ml; Corning) and DNaseI (250 μg/ml; Worthington Biochemical). Digested tissues were red cell lysed, filtered through a 70 μm nylon cell strainer (BD Biosciences), resuspended in PBS with 5% donor calf serum, 10 μg/ml Fc Block (2.4G2; Trudeau Institute) and anti-CD45 MACS beads (Miltenyi Biotec) on ice for 10 min. Cell suspensions were depleted of CD45-expressing cells using LS columns (Miltenyi Biotec) according to the manufacturer's instructions. The flow through cells were stained with anti-CD31 (390; Invitrogen); anti-CD45.2 (104) and anti-CD326 (EpCAM; G8.8; all from Biolegend) and anti-Sca-1 (Ly6A/E; D7; Invitrogen). Lung epithelial cells were sorted with FACSAria II (BD Biosciences) located at University of Alabama at Birmingham Flow Cytometry Core Facility.

### BAL Fluid Collection and Differential Cell Counts

Lungs were instilled with 3 ml Hank's balanced salt solution (HBSS; Corning) containing 0.05 mM EDTA (Lonza) and cell suspensions (≈10,000 cells in 100 μl) were centrifuged at 800 rpm for 5 min in a Shandon CytoSpin 3 cytocentrifuge (Cell Preparation System). The cytospin pellets were air dried on glass slides, stained with Shandon Kwik–Diff™ Stain kit (Thermo Scientific) and were mounted with Richard–Allan Scientific™ Cytoseal™ 60 (Thermo Scientific).

### Enzyme–Linked Immunosorbent Assay (ELISA)

Total IgE levels were determined by coating plates with 1 μg/ml purified rat anti–mouse IgE (R35–72; BD Biosciences). Standard curve was prepared with purified mouse IgE κ (C38–2; BD Biosciences) and bound Abs in serum samples were detected with biotin rat anti–mouse IgE (R35–118; BD Biosciences) and streptavidin–alkaline phosphatase (Invitrogen). Alkaline phosphate substrate (Moss, Inc.) was added, and color development was detected with SpectraMax® M2 (Molecular Devices) at 405 nm.

### Histology and Immunofluorescence

Paraffin–embedded sections (5 μm in thickness) were stained with hematoxylin and eosion (H&E) for standard histology and Periodic acid–Schiff (PAS) for airway mucus production. Frozen sections (8 μm in thickness) were prepared from lungs perfused with a mixture of optimum cutting temperature (OCT) compound (Sakura Finetek) in PBS at a 1:1 ratio. Sections were fixed in cold acetone for 10 min and blocked with Fc Block (10 μg/ml) and 5% (vol/vol) normal donkey serum in PBS at 25°C for 30 min. Sections were then stained with the following primary Abs in PBS at RT for 30 min: goat anti–CD3ε (M−20; Santa Cruz Biotechnology), anti–CD11c (HL3), and anti–CD45R (B220; RA3–6B2; all from BD Biosciences); anti–CD21/CD35 (7E9; BioLegend); and peanut agglutinin (PNA; Sigma). Primary Abs were detected with donkey anti–goat–Alexa Flour® 568, rabbit fluorescein–Alexa Fluor® 488, streptavidin–Alexa Fluor® 555, and streptavidin–Alex Flour® 488 (all from Invitrogen) at RT for 30 min. Slides were mounted with SlowFade® Gold Antifade Mountant with 4′,6–diamidino−2–phenylindole (DAPI; Invitrogen). Images were collected with a Zeiss Axiocam digital camera (Zeiss) or Nikon Andor Clara camera (Nikon). The images were obtained with a 20x objective for a final magnification of x200. T cells in immunofluorescent images were quantified by counting CD45.1^+^ cells in iBALT areas (defined manually using the outline tool) and by counting CD45.1^+^ cells in non–iBALT areas (excluding large empty airways). The mean number of T cells per μm^2^ was determined using a mosaic of images obtained from control and iBALT–containing lungs that were sectioned at a similar depth and orientation. Data were obtained from multiple sections from each mouse and 5 mice per group.

### RNA Isolation and Quantitative Real–Time PCR (qPCR)

Total RNA was extracted from lungs with TRIzol according to the manufacturer's specifications (Invitrogen) and was repurified with an RNeasy mini kit (Qiagen). Ribonucleic acid (RNA; 2.2 μg) was reverse–transcribed with Superscript II and random primers (Invitrogen) and complementary deoxyribonucleic acid (cDNA; 25 ng) was amplified with following primers and probes: *glyceraldehyde*−*3–phosphate dehydrogenase* (*Gapdh*; Trudeau Institute), *Il4, Il5, Il13, Chi3l4, Muc5ac*. *Tnfaip3*, and *Tslp* (all from Applied Biosystems), with TaqMan® Gene Expression Master mix (Applied Biosystems) and all reactions were run on a Lightcycler 480 Real–time PCR System (Roche). The relative level of messenger RNA (mRNA) expression for each gene was first normalized to the expression of *Gapdh* and then compared to the average level of mRNA expression in lungs from naïve B6 mice. Data is expressed as logarithmic fold changes in mRNA expression.

### Quantifying Cytokines in BAL Fluid

Lungs were instilled with 1 ml HBSS (Corning) containing 0.02 mM EDTA (Lonza) and SIGMA*FAST*™ Protease Inhibitor Cocktail Tablets (Sigma). Total protein levels of cytokines IL−4, IL−5, and IL−13 in lavage were quantified by mouse–specific Milliplex® multi–analyte kits (EMD Millipore) using a MagPix® instrument platform and related xPONENT® software (Luminex Corporation). The readouts were analyzed with the standard version of the Milliplex Analyst software (EMD Millipore).

### Statistical Analysis

The difference in mean values between two groups was analyzed with a two–tailed Student's *t*–test. If three or more groups were compared, Tukey's multiple comparison test was used (GraphPad Prism Version 6.0). *P*-values of less than 0.05 were considered statistically significant.

## Data Availability Statement

The raw data supporting the conclusions of this article will be made available by the authors, without undue reservation.

## Ethics Statement

The animal study was reviewed and approved by Institutional Animal Care and Use Committee, University of Alabama at Birmingham.

## Author Contributions

JH, AS-S, DC, MG-H, and JR-M designed and performed experiments. JH and TR wrote the manuscript. TR helped design experiments and obtained funding for the work. All authors contributed to the article and approved the submitted version.

## Conflict of Interest

The authors declare that the research was conducted in the absence of any commercial or financial relationships that could be construed as a potential conflict of interest.
