# Supplementary material for: Inducible Bronchus–Associated Lymphoid Tissue (iBALT) Attenuates Pulmonary Pathology in a Mouse Model of Allergic Airway Disease
Source: Front Immunol. 2020 Sep 25;11:570661. doi: 10.3389/fimmu.2020.570661 (PMC7545112; doi:10.3389/fimmu.2020.570661)
Supplement: Supplementary file 1 [file Data_Sheet_1.PDF]

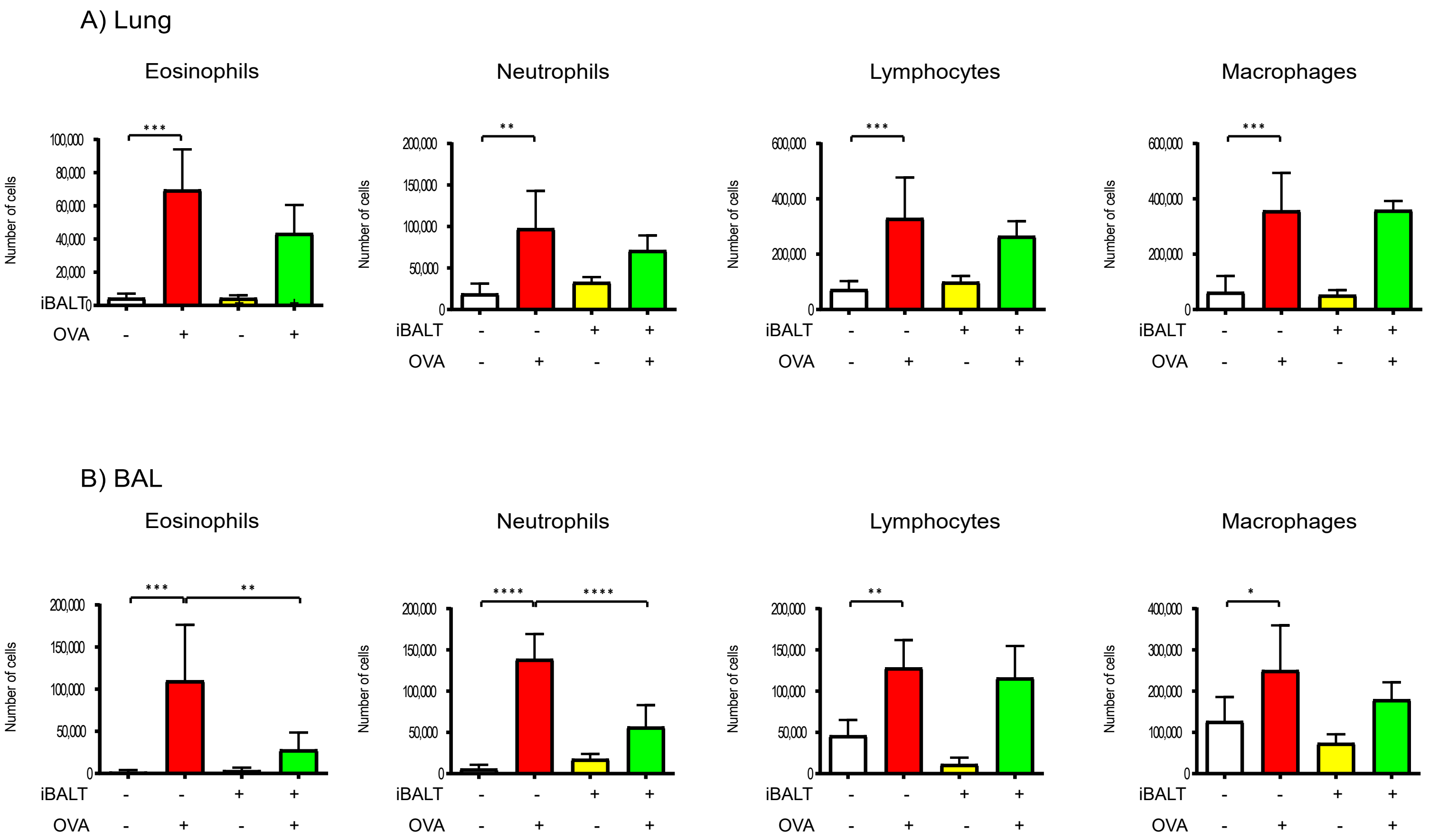

Supplementary Figure 1. The presence of iBALT reduces OVA–induced eosinophilia. The timing of iBALT induction, allergic sensitization and antigen challenge and analysis is shown in Fig 1E. Cells obtained from lung and BAL were counted and analyzed by cytospin to determine the proportion of neutrophils, lymphocytes, and macrophages/monocytes. The total number of each cell type is shown in the lung (A) and BAL (B). All data show mean ± SD of 4–5 mice per group; *P<0.05, **P<0.01, ***P<0.001, ****P<0.0001. Experiments were performed 4 times.
